# Very early remission and increased apoptosis with the use of Pentoxifylline in children with acute lymphoblastic leukemia

**DOI:** 10.3389/fonc.2024.1401262

**Published:** 2024-10-03

**Authors:** Violeta Salceda-Rivera, Pablo C. Ortiz-Lazareno, Georgina Hernández-Flores, Jorge R. Vazquez-Urrutia, Jesus Meza-Arroyo, Monzerrat Pardo-Zepeda, Hugo Romo-Rubio, Cesar Barba-Barba, Fernando Sánchez-Zubieta, Carlos Alfredo Barrón-Gallardo, Oscar Gonzalez-Ramella, Alejandro Bravo-Cuellar

**Affiliations:** ^1^ Immunology Division, Western Biomedical Research Center, Mexican Social Security Institute, Guadalajara, JAL, Mexico; ^2^ Doctoral Program in Biomedical Sciences, Centro Universitario de Ciencias de la Salud, Universidad de Guadalajara, Guadalajara, JAL, Mexico; ^3^ Department of Pediatric Hemato-Oncology, Hospital Civil de Guadalajara “Dr. Juan I. Menchaca”, Guadalajara, JAL, Mexico; ^4^ Centro Universitario de Ciencias de la Salud, School of Medicine, Universidad de Guadalajara, Guadalajara, JAL, Mexico; ^5^ Departamento de Clinicas de Reproduccion Humana, Crecimiento y Desarrollo Infantil, Centro Universitario de Ciencias de la Salud, Universidad de Guadalajara, Guadalajara, JAL, Mexico; ^6^ Departamento Académico de Disciplinas Especializantes de Ciencias de la Salud, Universidad Autonoma de Guadalajara, Zapopan, JAL, Mexico; ^7^ Centro Universitario de los Altos, Universidad de Guadalajara, Tepatitlán de Morelos, JAL, Mexico

**Keywords:** MRD, ALL, clinical trials, apoptosis, Pentoxifylline, early remission, childhood, treatment response

## Abstract

**Introduction:**

Despite the improvement in survival in acute lymphoblastic leukemia (ALL), there are still cases with evasion of chemotherapy-induced apoptosis. The IKK/NF-κB signaling pathway contributes to antiapoptotic gene expression. Pentoxifylline (PTX) inhibits IkB phosphorylation, blocking NF-κB and antiapoptotic activity.

**Methods:**

We conducted a randomized, double-blind clinical trial on pediatric ALL patients undergoing induction therapy, assigning them to PTX or placebo group. Bone marrow aspirates were obtained on days 1, 8, 15, and 22. Apoptosis was assessed using Annexin-V/propidium iodide.

**Results:**

Results indicated that the PTX group exhibited higher apoptosis on day-8 (41.3% vs. 19.4%, *p* =0.029) and day-15 (35.0% vs. 14.2%, *p* <0.01). On day-8, the PTX group displayed an MRD of 0.25% vs. 18.2% (*p* <0.01) in placebo group; on day-15, the PTX group demonstrated an MRD of 0.09% vs. 1.4% (*p* =0.02). Patients achieving an MRD <0.01% on day-8 demonstrated a 3-year Overall Survival (OS) of 81.6% vs. 58.3% (*p* =0.03); on day-15, patients with MRD <0.01% had a 3-year OS of 77.9% vs. 54.5% (*p* =0.03). The PTX group achieved an MRD of <0.01% earlier on days-8 and 15, along with a higher apoptosis rate, indicating a more favorable therapeutic response. In the entire cohort, patients achieving MRD <0.01% on day-8 or 15 displayed superior OS.

**Conclusion:**

Our study demonstrates that PTX enhances apoptosis and reduces MRD in pediatric acute lymphoblastic leukemia patients.

**Clinical trial registration:**

https://clinicaltrials.gov/, identifier NCT02451774.

## 1 Introduction

In recent years, a higher survival rate has been achieved in children with Acute Lymphoblastic Leukemia (ALL) due to scientific advances and the impact of new chemotherapy protocols (1). In children under 15 years of age, the 5-year Overall survival (OS) rate has increased from 60% in 1975 to 90% in 2005 (1, 2).

Initial treatment of patients with newly diagnosed ALL is based on a combination of chemotherapies. The intensity of chemotherapy is based on the group risk assessment. Traditionally, risk stratification has been based on clinical factors such as age and sex, white blood cell count at diagnosis, and immunophenotypic, cytogenetic, and molecular characteristics. The objective was to select the least possible toxic treatment according to the possibility of survival (2, 3).

In addition to the risk group, the percentage of minimal residual disease (MRD) at the end of induction is related to the long-term outcome (4). The treatment response is strongly influenced by the sensitivity or resistance of leukemic cells to drugs, as well as by the pharmacodynamics and pharmacogenomics of each patient; the early response has great prognostic importance (5–7).

The MRD results at the end of induction therapy or consolidation can change the prognosis assigned by the risk classification at the beginning of the treatment and is a tool used to define post-induction therapy (8). The majority of bone marrow MRD-based survival analyses have employed the value at the end of induction or consolidation therapy (9, 10). The impact of the treatment response during the initial days of induction therapy has not been widely studied (11).

Different types of chemotherapy are known to induce cellular stress through different mechanisms, resulting in leukemic cell apoptosis (12, 13). Resistance to cell death or apoptosis is one of the factors involved in cancer development and constitutes one of the factors in chemotherapy treatment to which a patient does not respond adequately (14).

Transcription factor NF-κB is involved in diverse regulatory functions of the response to cellular stress, proliferation, differentiation, apoptosis, and tumorigenesis (15). This transcription factor is constitutively activated in different types of cancer, including ALL, and has been associated with malignant cell survival and resistance to chemotherapy (16).

Pentoxifylline (PTX) can inhibit the phosphorylation of the NF-kB inhibitor (IkBa); this phosphorylation releases NF-kB for its subsequent translocation to the nucleus (17). Our group and others have proven that PTX possesses anti-tumor properties *in vivo* and *in vitro* (18–20). Even in children with ALL, PTX increases apoptosis, during the steroid prophase, by modifying the gene expression related to cell death and apoptosis (21, 22).

## 2 Materials and methods

### 2.1 Sample size

The sample size was determined based on data from a pilot study during the pre-phase of steroid induction to remission in pediatric patients with ALL (21). This study aimed to detect significant differences in the mean apoptosis between the placebo and the PTX groups, in a population where the standard deviation was known. Using the formula for comparing means with known standard deviations, we calculated the necessary sample size to achieve a 95% confidence level and 80% power. The analysis indicated that a minimum of 22 patients per group would be required to reliably detect the expected differences in mean apoptosis.

### 2.2 Patients

Between the years 2014 and 2018, 44 consecutive pediatric patients (ranging in age from 3-17 years), with newly diagnosed ALL were enrolled in this randomized, double-blind phase 2 clinical trial (ClinicalTrials.gov, number NCT02451774). Patients must weigh 20 kg or more and must be able to swallow, due to the dose of placebo and PTX, and the fact that the splitting of tablets was not allowed. All patients were treated with the Total Therapy XV chemotherapy scheme according to their risk group. The patients were randomly assigned to two treatment groups of 22 patients each. Thus, during induction therapy, PTX was added to one group, and placebo was added to the other. Both the Ethics and Research Committees approved this clinical trial. Written informed consent was obtained from the children’s parents or guardians.

Risk classification was based on Total Therapy XV therapy-scheme risk-group criteria. Patients were considered low-risk if they met each of the following criteria: B-ALL immunophenotype (excluding mature B cell immunophenotype); age between 1 and 10 years; leukocyte count <50 x 10^9^/L at diagnosis; a leukemic cell DNA index of ≥1.16 (or hyperdiploidy >50); t(12;21)/(ETV6-RUNX1)-positive, and lack of CNS3 status or testicular leukemia. High-risk ALL was considered if patients presented any of the following criteria: BCR-ABL1; T-ALL immunophenotype; CNS3 status, age <1 or ≥10 years, and a leukocyte count of ≥50 x 10^9^/L at diagnosis. If the patients did not meet all the criteria for low-risk and did not present any high-risk characteristics, they were classified as standard-risk.

### 2.3 Treatment

We used the Total Therapy XV chemotherapy scheme, with a steroid prophase for 1 week, employing Prednisone calculated at 40 mg/m^2^/day orally, corresponding to days 1 to 7, and the four drugs were started on day 8. In this clinical trial, bone marrow aspirates were performed on days 1 (at diagnosis), 8 (at end of steroid prophase), 15, and 22 to evaluate response to therapy (MRD), apoptosis, and senescence, as described in **Table 1**. None of the patients included in this clinical trial received bone marrow transplant.

**Table 1:** Induction therapy based on Total Therapy XV.

Agents	Doses	Schedules
Steroid prophase (prednisone alone)	40 mg/m^2^/day PO (tid)	Day 1-7
Prednisone	40 mg/m^2^/day PO (tid)	Day 1-35
Vincristine	1.5 mg/m^2^/week IV	Day 8, 15, 22, 29
Daunorubicin	25 mg/m^2^/week IV	Day 8, 15
L-asparaginase	10 000 U/m^2^/dose IM	Day 9, 11, 13, 15, 17, 19
Cyclophosphamide	1 000 mg/m^2^/dose IV	Day 29
Cytarabine	75 mg/m^2^/dose IV	Day 30-33, 37-40
6-Mercaptopurine	60 mg/m^2^/dose PO	Day 29-42
Bone marrow evaluations (MRD*, apoptosis, and senescence assays)	Weekly	Day 1 (diagnosis), 8, 15, and 22
Pentoxifylline	Weight-based	Day 1-28
Placebo	Weight-based	Day 1-28

PO, taken by mouth, Per Os.

tid, three times a day (from Latin ter in die).

IV, intravenous.

IM, intramuscular.

MRD, minimal residual disease.

MRD*, Minimal residual disease was only performed on days 15 and 22.

The dose of PTX was calculated based on the patient’s weight; 400 mg tablets were utilized according to the following weight categories: patients weighing 20-25 kg received one tablet once daily; those with a weight of 26-40 kg received one tablet twice daily, and patients weighing >41 kg received one tablet three times daily. Crushing or splitting pills was not allowed; thus, the patients were required to have swallowing capacity. The number of daily placebo tablets was based on the patient’s weight category.

### 2.4 Sample preparation

Bone Marrow Mononuclear Cells (BMMC) were obtained using density gradient isolation (Ficoll-Paque™ Plus; GE Healthcare, Uppsala, Sweden). The obtained BMMC was utilized for apoptosis and senescence assays and for flow cytometry MRD.

### 2.5 Apoptosis assay

From BMMC, apoptosis was evaluated using the Annexin-V-Flous Staining commercial kit (Roche Molecular Biochemicals, Indianapolis, IN, USA), according to the manufacturer’s instructions. The samples were analyzed by flow cytometry. At least 20,000 events were acquired for each sample in a FACSAria I Cell Sorter (BD BioScience, San Jose, CA, USA), and the data analysis was performed employing Kaluza Analysis Software Ver.2.1.

Cells that were Propidium iodide (PI). and Annexin-V negative were considered live cells. Cells that were PI-negative and Annexin-V-positive were considered in early apoptosis. Cells that were positive for both PI and Annexin-V were considered in late apoptosis, and only PI-positive cells were considered necrotic. Data obtained from early and late apoptosis were handled together as apoptosis in the Results section. The results are shown as mean ± standard deviation (SD) of the percentage of apoptotic cells.

### 2.6 Senescence assay

Senescence determination was made by measuring β-galactosidase activity (Senescence-Associated beta-gal [SA-β-gal]). BMMC cells (1 x 10^6^) were obtained; these were diluted in 250 µl of PBS. Then 100 nM of Bafilomycin A1 (Sigma Aldrich, B1793, *Streptomyces griseus* Bafilomycin A1) was added, and the cells were incubated for 1 h at room temperature.

Subsequently, 10 μM of C12FDG (Fluorogenic glycosidase substrate; Invitrogen Corp., Carlsbad, CA, USA) was added, and the mixture was incubated for 15 min. Finally, the cells were harvested, washed twice with PBS, and resuspended in PBS before being analyzed by flow cytometry. The data analysis was performed employing Kaluza Analysis Software Ver. 2.1. The results are shown as mean ± SD of the percentage of senescent cells.

### 2.7 Minimal residual disease assessment by multicolor flow cytometry

Leukemia-associated immunophenotypes were analyzed by 10-color flow cytometry (Beckman Coulter Gallios^®^), with Beckman Coulter reagents and antibody labeling protocols. CD34-FITC/CD19-PE/CD45-PerCP/CD22-APC and TdT-FITC/CD19-PE/CD20-PerCP/CD10-APC were used in patients with BCP-ALL, while in patients with T-ALL, the labeling TdT-FITC/CD7-PE/CD19-PerCP/CD3-APC, CD2-FITC/CD7-PE/CD3-PerCP/CD5-APC and CD7-FITC/CD16.56-PE/SmCD3-PerCP/CyCD3- APC were utilized. We aimed for 1 million cells per tube. All MRD data are expressed as the percentage of MRD within the leukocytes. We utilized a result of <0.01% of malignant cells to consider whether the patient was in remission.

### 2.8 Statistical analysis

The Shapiro-Wilk normality test was used. For non-parametric variables, data are presented as mean ± SD, and *p* values were determined by the Mann-Whitney *U* test. For categorical variables, the Fisher exact test was used. For correlation, the Phi coefficient was employed. OS rates were estimated by the Kaplan-Meier method and compared by the log-rank test, *p <*0.05 was used as the threshold for statistical significance. IBM SPSS Statistics ver. 26 was used for the statistical analyses.

## 3 Results

### 3.1 General characteristics of patients

**Figure 1:**
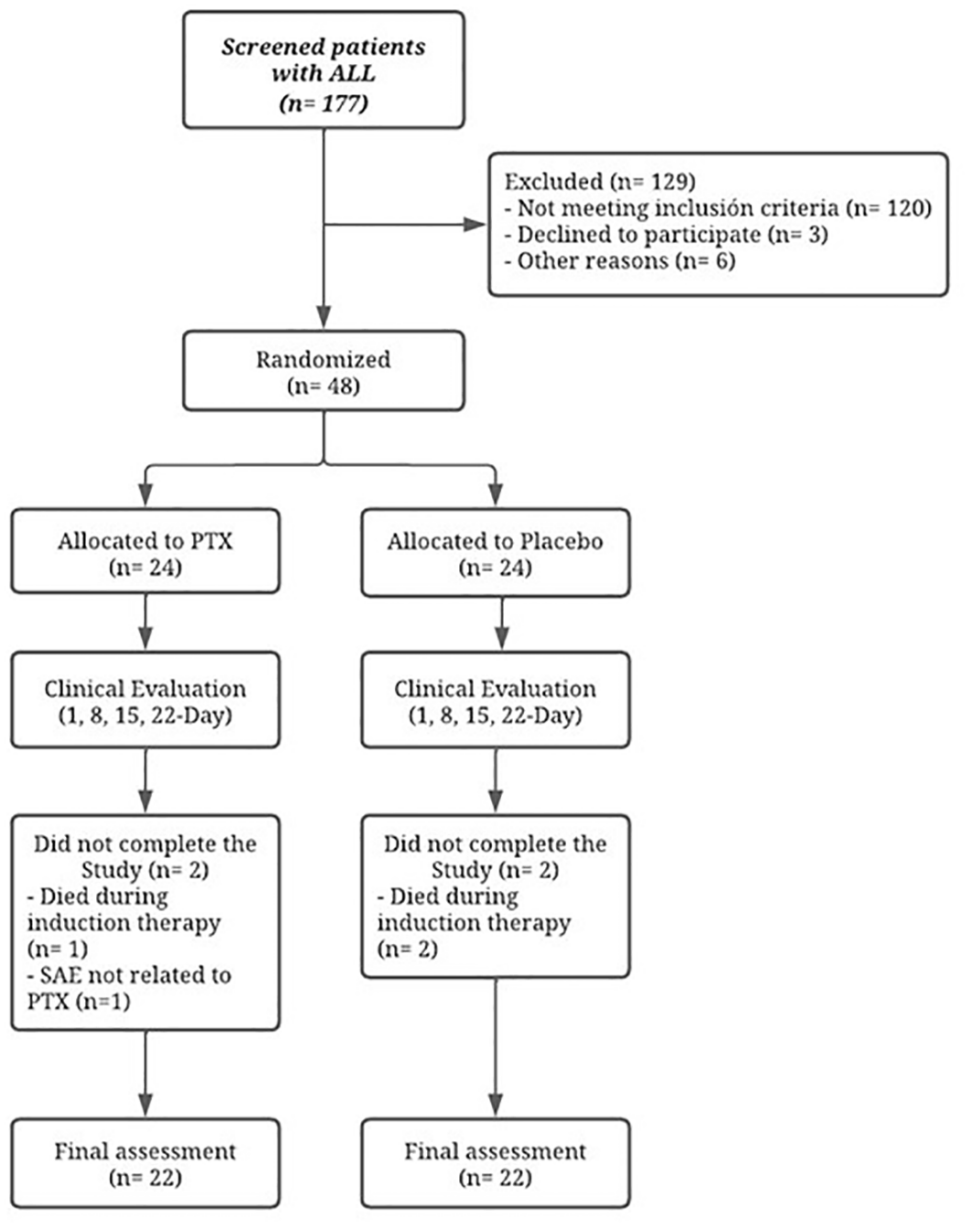
Flow diagram of the study. PTX, pentoxifylline; SAE, serious adverse event.

A total of 44 pediatric patients with ALL, diagnosed from the years 2015-2018, were included as shown in **Figure 1**. The average age of both groups was 10.6 ± 3.53 years (range 3-17). A total of 86.3% (*n* = 38) of the patients were classified as high- or standard-risk, and 13.6% (*n* = 6) met all criteria for the classification of low-risk patients. Leukocyte count at the time of diagnosis was 46.2 x 10^3^/mm^3^, with a range of 0.89-279.02 x 10^3^/mm^3^. Only six patients had T-cell immunophenotype; the rest had B-cell precursor phenotype. Our risk ratio and average age may have been influenced by the minimal weight of 20 kg and swallowing capacity as inclusion criteria, which gave us a high proportion of patients older than 10 years of age. The demographic characteristics of the patients by treatment group are analyzed in **Table 2**.

**Table 2:** Clinical-biological characteristics of the patients by treatment group.

	Pentoxifylline	Placebo	*P* value
No. cases	22	22	NS
Age			
Years (mean ± SD) Range	10.6 ± 2.9 6-15	10.68 ± 4.1 3-17	NS
Gender			
Masculine Feminine	10 (45.5%) 12 (54.5%)	13 (59.1%) 9 (40.9%)	NS
Risk group			
High-risk Standard-risk Low-risk	16 (72.7%) 3 (13.6%) 3 (13.6%)	16 (72.7%) 3 (13.6%) 3 (13.6%)	NS
Leukocytes at diagnosis			
10^3^/mm^3^ (mean ± SD)	43.2 ± 74.4	49.1 ± 52.5	NS
Immunophenotype			
B-cell T-cell	19 (86.4%) 3 (13.6%)	19 (86.4%) 3 (13.6%)	NS

SD, standard deviation.

NS, not significant.

### 3.2 Determination of apoptosis in patients with ALL according to treatment group

**Figure 2:**
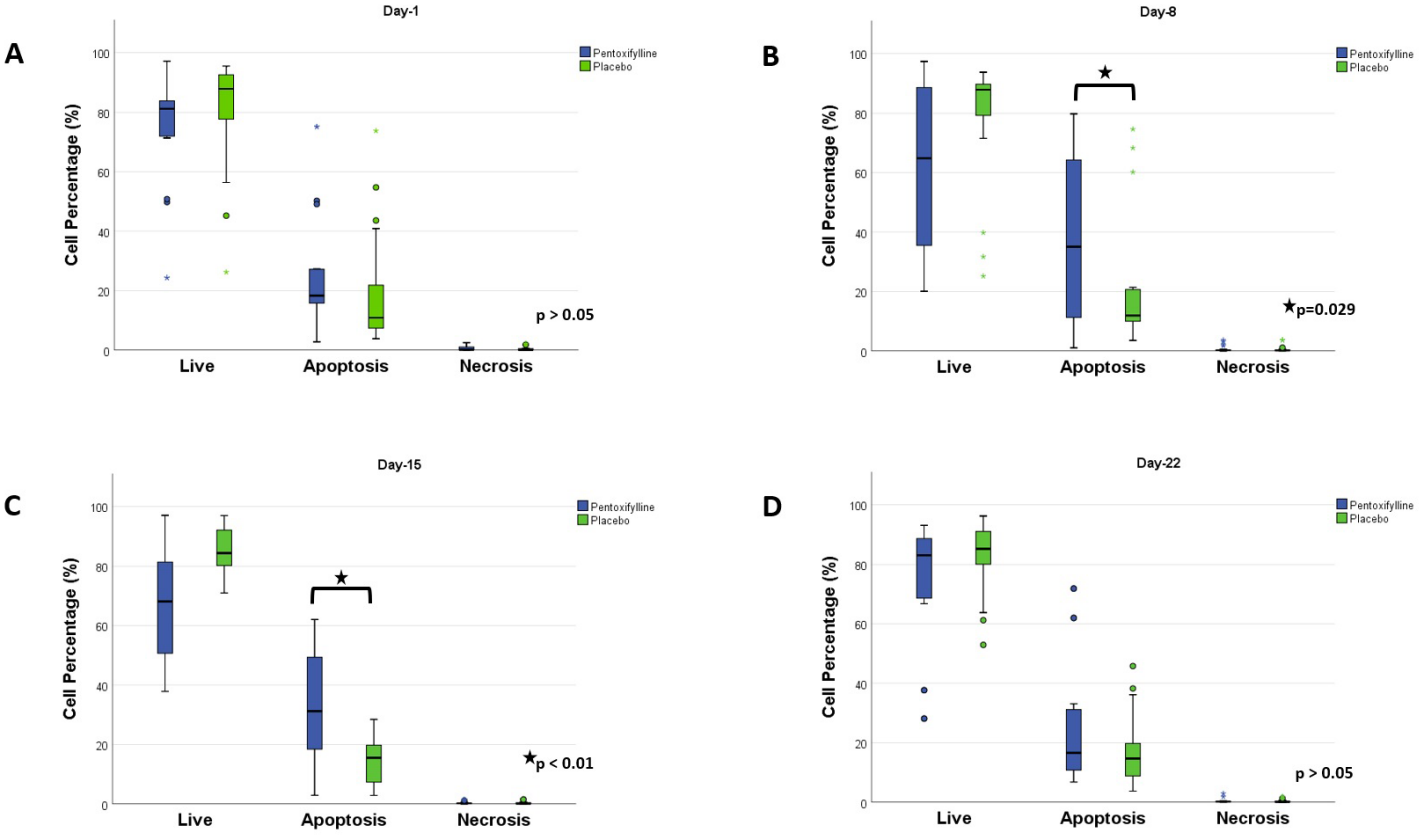
Apoptosis comparison between the two treatment groups. Flow cytometry was performed using Annexin V-Fluorescein isothiocyanate (FITC)/Propidium iodide (PI) to determine if the cells were live, apoptotic, or necrotic. **(A)** Day 1. No difference was observed. **(B)** Day 8. The PTX group shows a significantly higher percentage of apoptotic cells. **(C)** Day 15. The PTX group shows a significantly higher percentage of apoptotic cells. **(D)** Day 22. No differences were found between the groups. Statistical analysis, Mann–Whitney U test, considering p<0.05 as significant.

In both treatment groups, apoptosis of the BMMC was determined by flow cytometry. No significant differences were found at the time of diagnosis (day 1) nor on day 22 of induction therapy. However, on day 8, when the steroid prophase ends and combined chemotherapy starts, we found, in the PTX group, an apoptosis rate of 41.3% ± 29.9, compared to 19.4% ± 20.3 presented in the placebo group (*p* = 0.029). We found a significant difference on day 15, in the PTX group, the apoptosis rate was 35.0% ± 18.1, compared to 14.2% ± 7.5, as presented in the placebo group (p <0.01). Regarding necrosis, no significant differences were found between the treatment groups, as shown in **Figure 2**.

### 3.3 Determination of senescence in patients with ALL according to treatment group

**Figure 3:**
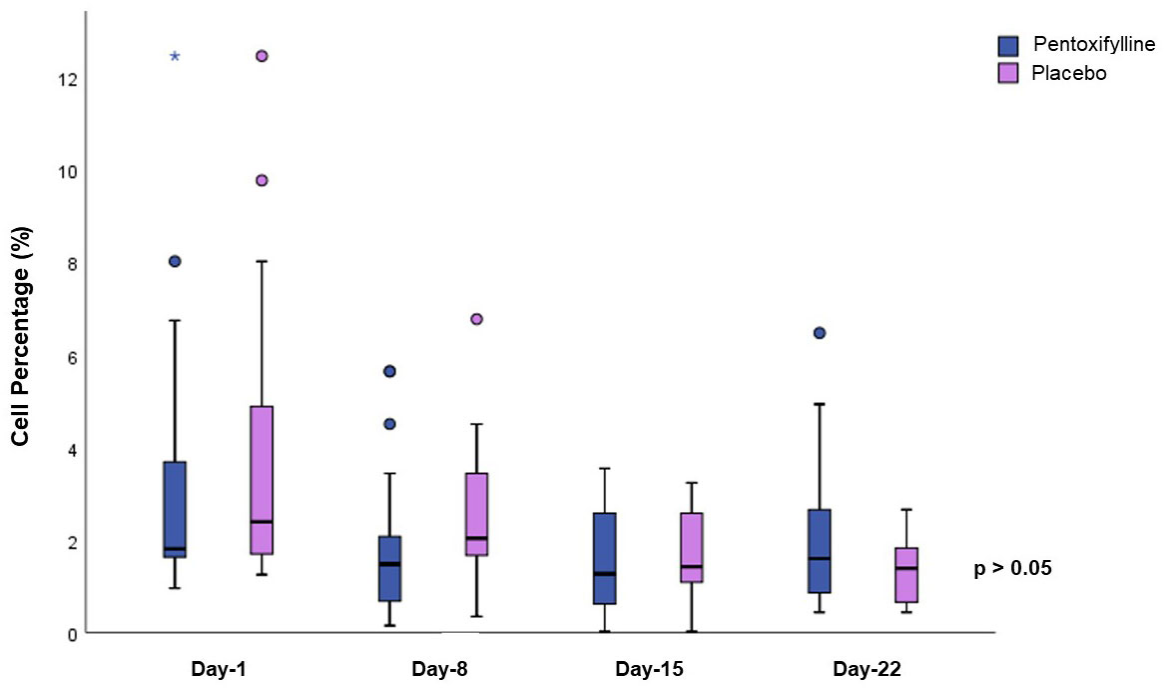
Senescence comparison between the two treatment groups. Determination of senescence was made by measuring β-galactosidase activity by flow cytometry. No differences were found between the groups. Statistical analysis, Mann–Whitney U test, considering p<0.05 as significant.

Throughout the study, the senescence was maintained with similar percentages, we did not observe a significant difference between the treatment groups, as shown in **Figure 3**.

### 3.4 Minimal residual disease results

On day 15, in the PTX group, 86.3% (*n* = 19) of the patients were in remission (MRD <0.01%), the placebo group showed 31.8% (*n* = 7) of patients in remission (*p <*0.01). In the PTX group, we found an MRD of 0.25% ± 0.18, compared to the placebo group, with an MRD of 18.2% ± 6.6 (*p <*0.01).

On day 22, a total of 90.9% (*n* = 20) of the patients from the PTX group were in remission, compared to 59.1% (*n* = 13) of the patients in the placebo group (*p* = 0.034), the PTX group patients showed an MRD of 0.09% ± 0.07, while patients in the placebo group presented an MRD of 1.4% ± 1.1 (*p* = 0.02).

To determine whether the MRD on day 15 was associated with the treatment group, we obtained a Phi correlation coefficient of 0.555, indicating that there is a moderate-to-strong association between presenting an MRD of <0.01% with the use of PTX (*p <*0.01). However, with respect to the MRD on day 22, we obtained a Phi correlation coefficient of 0.367, indicating a low association of an MRD of <0.01% and the use of PTX (*p* = 0.015). The latter findings are summarized in **Table 3**.

**Table 3:** Minimal residual disease by treatment group.

	Pentoxifylline *n* = 22	Placebo *n* = 22	*P* value
MRD-negative on day 15 Yes No	19 (86.4%) 3 (13.6%)	7 (31.8%) 15 (68.2%)	0.001^a^
MRD-negative on day 22 Yes No	20 (90.9%) 2 (9.1%)	13 (59.1%) 9 (40.9%)	0.03^a^
% MRD on day 15 (mean ± SD)	1.06 ± 3.9	18.2 ± 31.3	0.001^b^
% MRD on day 22 (mean ± SD)	0.07 ± 0.3	1.4 ± 5.4	0.02^b^
Association of remission on day 15 with treatment group	Moderate-to-strong correlation coefficient 0.55^c^	Moderate-to-strong correlation coefficient 0.55^c^	0.001^a^

Statistical significance tests: ^a^Fisher exact test; ^b^Mann-Whitney U test; ^c^Phi coefficient.

SD, Standard Deviation.

### 3.5 Survival in patients with ALL based-on treatment group and MRD results

**Figure 4:**
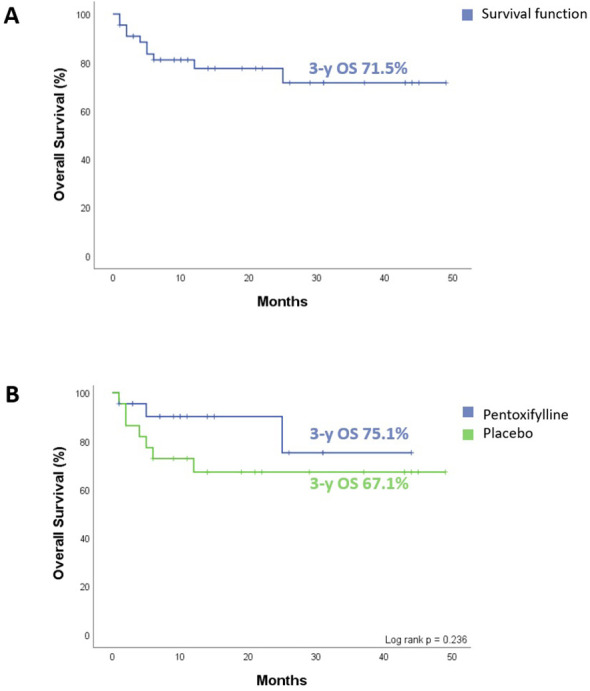
Overall survival (OS) of all patients enrolled in the clinical trial. **(A)** The entire cohort 3-year OS. **(B)** Comparative 3-y OS between the two treatment groups, no difference observed (p = 0.236). Statistical analysis, log-rank test, considering p <0.05 as significant.

The entire cohort presented a 3-year OS of 71.5% (95 CI: 56.7% to 90.2%). When comparing survival by treatment group, we found no significant differences. For patients in the PTX group, the 3-year OS was 75.1% (95 CI: 51.1% to 99.1%). For those in the placebo group, the 3-year OS was 67.1% (95 CI: 49.7% to 90.6%), (*p* = 0.236). This is represented in **Figure 4**.

**Figure 5:**
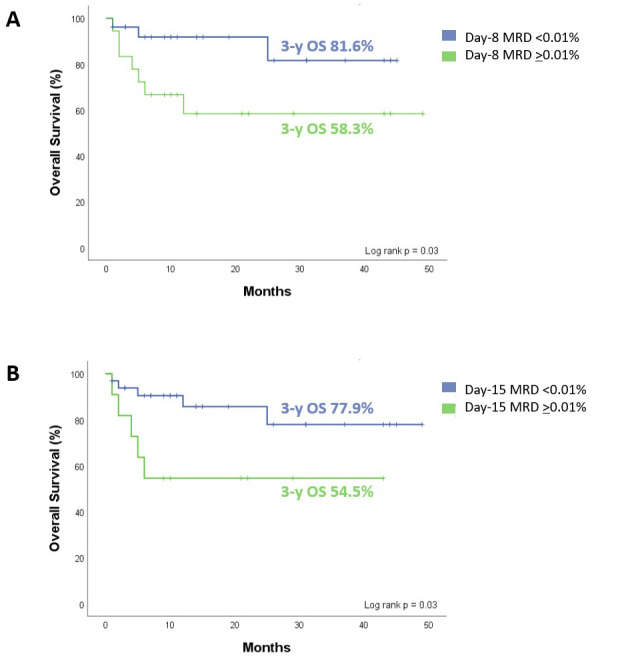
Overall survival (OS) of all patients enrolled as a function of level bone marrow MRD. **(A)** Day 8. MRD ≥0.01% with 3-y OS of 58.3%, and MRD <0.01% of 81.6% (p = 0.03). **(B)** Day 15. MRD ≥0.01% with 3-y OS of 54.5%, and MRD <0.01% of 77.9% (p = 0.03). Statistical analysis, log-rank test, considering p<0.05 as significant.

Knowing that treatment response is considered an independent prognostic factor and that the PTX group achieved complete remission earlier, we analyzed OS in the entire cohort based on treatment response on day 8. We found that in the patients who, on day 8 presented an MRD of ≥0.01%, the 3-year OS was 58.3% (95 CI: 38.4% to 88.7%). Patients with an MRD of <0.01% had a 3-year OS of 81.6% (95 CI: 62.9% to 99.0%), (*p* = 0.03), as depicted in **Figure 5**.

We also analyzed the impact of the MRD result on day 15 in the entire cohort. Patients who had an MRD of ≥0.01% had a 3-year OS of 54.5% (95 CI: 31.8% to 93.6%). Those patients with an MRD of <0.01%, the 3-year OS was 77.9% (95 CI: 61.1% to 99.3%), (*p* = 0.03), as presented in **Figure 5**.

Of the ten deaths that occurred, seven were due to treatment complications, and three were due to disease progression; two of these patients were in the placebo group. All patients classified as low-risk in both groups survived and did not experience relapse.

### 3.6 Adverse effects related to PTX

During this clinical trial, no patient exhibited adverse effects related to PTX.

## 4 Discussion

Acute lymphoblastic leukemia (ALL) is the most frequent neoplasm in childhood and cure rates of over 90% have been achieved in developed countries; despite therapeutic advances in many countries, this survival rate has not yet been achieved in all cases (23). Efforts are needed to reduce treatment-related toxicity, improve early detection, and prevent treatment abandonment, among other aspects. Targeted therapy with novel drugs could be a strategy to increase treatment success in areas where the cure of patients with ALL remains low.

In this study, we evaluated the addition of PTX to standard ALL treatment during induction therapy to achieve earlier remission and investigated its direct effect on tumor cell apoptosis. Published research has shown that PTX inhibits the phosphorylation of the NF-kB inhibitor: IKB-alpha (17, 18). NF-kB is widely recognized for its role in cell survival, proliferation, and the modulation of gene expression associated with apoptotic pathways (22).

The results of this study demonstrated that the administration of PTX during induction therapy significantly increased the percentage of apoptosis in BMMC (see **Figure 2**). These findings are consistent with those of a previous study conducted by our group in children with ALL during the steroid window (21). However, the latter study only investigated the effect of PTX on apoptosis after the first 7 days of treatment with steroids (day 8), prior to the start of intravenous chemotherapy. In contrast, in the present study, PTX was added during throughout the entire induction therapy to one group compared with another group that did not receive PTX; this allowed a more extended evaluation of the effect of PTX on tumor cell apoptosis and its potential benefit as an adjunct therapy in the treatment of ALL. We found that apoptosis continued to increase significantly on days 8 and 15; nonetheless, no differences were observed on day 22. An explanation is that on day 22 (after 2 weeks of induction therapy with four drugs), the majority of patients reached remission, as observed in the study by Pui et al., in which only 92 of 478 patients persisted with an MRD of >0.1% at day 19; these results are relevant because it is known that the earlier remission is achieved, the better the prognosis (24).

Although multiple studies have demonstrated the effectiveness of PTX in inducing apoptosis in tumor cells, it is noteworthy that these studies were conducted *in vitro* and reported a higher percentage of cells undergoing apoptosis than those observed in our patients (25). It is crucial to consider that clinical trials take place in a less controlled microenvironment and involve various factors, such as the mononuclear phagocytic system, which continuously eliminates apoptotic bodies (26), as well as the daily production of hematopoietic cells (27).

One discovery regarding senescence is that we observed in our study a consistently low level of senescent cells throughout the induction phase. This finding can be attributed to the nature of leukemia, a type of cancer characterized by rapid cell proliferation, and it is found in premalignant lesions or slow-replicating neoplasms where higher rates of senescence have been reported (28).

There is evidence suggesting that therapy-induced senescent cells may acquire a pro-tumor effect by escaping senescent arrest and re-entering a proliferative state and, also because the senescent cells release a secretome that may contain factors with pro-tumorigenic properties (29, 30). Those phenomena raise concerns about the long-term efficacy of treatment. A significant observation of this study is that PTX did not induce senescence: we observed consistently low levels of senescent cells throughout the induction therapy in our study. This finding can be attributed to the nature of leukemia, a type of cancer characterized by rapid cell proliferation, and it is found in premalignant lesions or slow-replicating neoplasms where higher rates of senescence have been reported (28).

In the context of ALL, MRD represents the most significant prognostic indicator. Typically, the analysis of treatment response using MRD is conducted at the end of induction therapy, as outlined in different chemotherapy protocols (7, 9, 10, 24). However, in our study, we opted to assess bone marrow MRD at an earlier stage, specifically after the first week of steroid treatment and following the initial dose of intravenous chemotherapy, corresponding to days 8 and 15. Only a few studies have explored the correlation between achieving very early remission at these specific time points and its impact on treatment outcomes, as described in the following studies (4, 10, 31).

The Children’s Oncology Group, as evidenced in a study conducted by Borowitz et al., revealed that patients with a peripheral-blood MRD level exceeding 1% on day 8 exhibited 5-year Event-Free survival (EFS) of 79%, whereas those with lower MRD levels achieved a 5-year EFS of 90%, even if they eventually tested negative for MRD by day 29 (4).

Similar to our findings, a study conducted by Loosveld et al. also demonstrated a more favorable prognosis in patients who tested peripheral-blood MRD-negative on day 15 (post diagnosis). This assessment was performed after the steroid prophase and following 1 week of chemotherapy. The study reported a significantly improved 4-year Disease-Free Survival (DFS) rate of 91.6% compared to 67.6% in patients who remained MRD-positive (*p* = 0.0013) (31).

Nevertheless, unlike our study, previous studies employed peripheral blood and not bone marrow to analyze treatment response. We employed bone marrow because we sought to increase sensitivity (32). As in our clinical trial, Basso et al. utilized bone marrow to perform MRD; these authors found that flow-cytometry bone-marrow MRD at day 15 was the most important prognostic factor, and they classified patients according to three risk groups based on the level of MRD at day 15: standard (<0.1%); intermediate (0.1 to <10%), and high (>10%); their 5-year cumulative relapse incidences were 7.5%, 17.5%, and 47.2%, respectively (11).

Our findings align with the previously reported results by our group, indicating that better survival outcomes were observed when patients achieved an MRD level of less than 0.01% on days 8 and 15 (post-diagnosis). Notably, this effect was particularly pronounced in patients who received PTX, as they exhibited significantly lower MRD levels on both days 8 and 15 compared to the group that did not receive the drug (see **Table 3**).

The cohort in our study exhibited a 3-year OS of 71.5%, similar to the 5-year OS reported in Mexico, ranging from 43.7%-74.7% according to risk, and with an average 5-year OS of 61.8% (33). Moreover, it is also similar to the survival rates reported in Latin-American countries, where 5-year net survival was still less than 70%, even after adjusting for very high background mortality in childhood: with in Brazil, Chile, Colombia, and Peru, and less than 60% in Mexico and Ecuador (23).

However, our results are considerably lower than the OS rate reported by St. Jude Children’s Research Hospital, which reported a 5-year OS of 93.5% using the same treatment protocol (34). It is essential to consider that Mexico belongs to middle-income countries, and our circumstances here are significantly different. Other countries in the same economic category as Mexico, which also implemented the same chemotherapy regimen, have reported low survival rates. For instance, a study by Chona de Armas et al. reported a 2-year OS of 57% and an EFS of 18.8% (35).

The overall conditions of our patients and their families, the nutritional status of the patients, and the limited availability of healthcare services in various areas contribute to increased morbidity and mortality from chemotherapy toxicity (36). Adjusting the treatment approach for children demonstrating a very early response could aid in reducing treatment-related toxicities. Indeed, tailoring treatment based on the patient’s response forms the foundation of the most innovative protocols (37).

In this clinical trial, most patients were classified in the high-risk group compared to other international publications (38, 40). We think that this difference is due to a conditioning factor in the selection of patients for the study; it was established as an inclusion criterion that patients have had a minimal weight of 20 kg and swallowing capacity since there was no presentation of suspension of the PTX, and the tablets could not be split or crushed due to pharmacological considerations. The former led to the inclusion of older patients, which increased the proportion of high-risk patients.

During the clinical trial, no patients experienced any adverse effects related to PTX. This finding leads us to consider PTX as a safe drug for administration in pediatric patients, which is consistent with results reported in other pediatric studies (39, 40).

However, it is essential to acknowledge that further research and larger-scale studies are necessary to validate these findings and to ensure the long-term safety of PTX administration in pediatric patients.

## 5 Conclusions

Chemotherapy exerts its primary action by causing irreparable cell damage through various pathways, predominantly inducing apoptosis as a mechanism of cell death. However, novel approaches in both *in-vitro* and *in-vivo* settings aim to enhance the effectiveness of chemotherapy, induce cell death, or halt proliferation using non-cytotoxic drugs to reduce the tumor burden before the emergence of resistance.

Increasing apoptosis while minimizing adverse effects holds significant potential for improving the survival outcomes of children with cancer. In this study, patients receiving a combination of PTX and chemotherapy achieved an MRD of <0.01% at an earlier stage. Early remission is known to be associated with better survival and serves as an independent prognostic factor. The favorable therapeutic response observed may be attributed to the substantial increase of apoptotic cells induced by PTX.

Based on the findings of the current clinical trial and in line with previous studies conducted in pediatric populations, PTX appears to be a safe therapeutic option for pediatric patients. The absence of reported adverse effects during this trial offers valuable insights and encourages further exploration of the potential benefits and safety of PTX in pediatric populations.

### Study limitations

In this study, apoptosis was measured in the entire population of bone marrow mononuclear cells to obtain a broader understanding of the treatment’s effects on the overall bone marrow microenvironment. Future studies should aim to refine this analysis by isolating leukemic blasts to delineate the specific apoptotic response in these cells.

## Data Availability

The raw data supporting the conclusions of this article will be made available by the authors, without undue reservation.
